# Memory compensation strategies in everyday life: similarities and differences between younger and older adults

**DOI:** 10.1038/s41598-023-34815-3

**Published:** 2023-05-24

**Authors:** Madeleine J. Radnan, Riley Nicholson, Ruth Brookman, Celia B. Harris

**Affiliations:** The MARCS Institute for Brain, Behaviour, and Development, Western Sydney University, Penrith, Australia

**Keywords:** Human behaviour, Psychology

## Abstract

Memory compensation strategies serve an important role in everyday functioning, especially in the face of cognitive decline. Research on the external memory compensation strategies employed by older adults has focused almost entirely on non-digital tools. Less is known about how memory compensation strategies might have changed due to the rapid and widespread uptake of digital technologies. In the current research, 208 younger adults and 114 older adults freely reported what internal or external memory strategy or tool they would use to accomplish 20 different everyday memory tasks. Participants’ responses were coded as involving either internal (e.g. using a mnemonic) or external (e.g. writing a list) strategies, and then underwent further categorisation to classify types of internal and external strategies (e.g. digital or physical tool). Findings indicated that external strategies were much more prevalent than internal strategies for both younger and older adults, and that digital compensation strategies were prevalent for both age groups. There were age differences such that older adults reported more strategies overall, and were less likely to report digital tools, more likely to report physical tools, more likely to report environmental tools, and less likely to report social tools than younger adults. Positive attitudes to technology were associated with digital tool use for older but not younger participants. Findings are discussed in terms of existing theories and approaches to studying memory compensation strategies and cognitive offloading.

## Introduction

A large literature focuses on the memory compensation strategies used by older adults, which include external tools like diaries and calendars to support day-to-day memory function^1–3^. “Compensation” refers to practices which people strategically employ to reduce the impact of cognitive impairments or mitigate the likelihood of cognitive failures^1,4^, and compensation has been particularly studied in the context of older age, cognitive decline, and neurological injury or impairment. That is, compensation behaviours arise from an objective or perceived deficit^1^, where there is a mismatch between expected and desired performance on a particular task. Compensation involves short-term reduction or elimination of the gap between desired and actual performance^5^, through increased cognitive effort or intensity^5^, or substitution with new reliable strategies that replace old strategies that are no longer functional^1^.

The extent to which people report engaging in memory compensation strategies is associated with individual characteristics. For instance, for older adults, those who are relatively older report using more compensatory strategies than those who are relatively younger; older women report making greater effort in remembering and using more internal and external strategies than do older men; older men report a greater frequency of relying on other people to remember than do older women; older people experiencing cognitive decline report more frequent use of compensation strategies including relying on others^6^ and dedicating more time and effort towards memory tasks compared to those who are not experiencing cognitive decline^2^; and older people reporting poorer physical and psychological health report more compensation strategies^7^. In addition to individual differences, memory compensation is also influenced by social and interpersonal factors. Harris et al. reported that the compensation strategies of partners within couples were associated with each other, such that partners were correlated in the extent to which they used external strategies including physical tools and reliance on others^8^. Overall, these findings suggest that both actual and perceived need for cognitive support influence the extent to which people adopt compensation strategies.

Although the literature on memory compensation has focused on people who are older or who have a cognitive impairment, other literatures suggest that using tools and strategies to augment individual cognition is a more general phenomenon. A relatively recent literature on ‘cognitive offloading’ has examined when and how people manipulate the environment to make cognitive tasks easier or more likely to succeed^9^. Cognitive offloading is a strategic process that occurs across the lifespan, even in young children^10^. People are particularly likely to spontaneously offload when tasks are more cognitively demanding^9^, consistent with the literature on memory compensation which emphasises that compensation occurs when people perceive that their performance on a particular task might be poor^1,5^. Schryer and Ross reported that a similar majority of both younger and older adults attempted to use a pen when given a phone message^11^. Taken together, these findings suggest that cognitive offloading strategies are employed lifelong for challenging tasks, and that different kinds of internal and external strategies may be better suited to different kinds of cognitive tasks^12^. In contrast with the positive framing of memory compensation strategies as a form of ‘offloading’ that boosts performance, research on digital memory tools in cognitive psychology has tended to be more pessimistic about the impact of such tools. For example, studies of “photo-impairment effects on memory” ^13^ or “Google effects on memory”^14^ both suggest that storing information externally impairs internal memory for that information, although there are mixed findings that make drawing conclusions problematic (for detailed review see Storm & Soares)^15^.

A few studies have directly compared younger and older adults in terms of their reported everyday memory compensation strategies. Bouazzaoui et al. reported that age was positively correlated with use of external memory strategies (all physical) and negatively correlated with use of internal strategies, suggesting that people increasingly outsource with age^16^. To examine differences in the types of external tools adopted, Finley et al. conducted a large survey of adults aged 18–75 regarding their use of physical and digital tools to complete memory tasks^17^. Across age groups, people reported the most frequent use of digital memory tools, slightly less frequent use of physical memory tools, and infrequent use of social memory supports. Finley et al. reported a correlation between physical tool use and age, such that older adults reported more frequent use compared to younger adults, but no corresponding negative correlation between digital tool use and age^17^. Finley et al. noted the shortcomings of brief Likert scales for capturing everyday memory compensation strategies, and called for more nuanced research investigating the tools and strategies that people actually use day-to-day^17^.

Therefore, despite the substantial literature on memory compensation strategies and individual factors that predict their use, there are a number of unanswered questions. The widely used Memory Compensation Questionnaire^3^, designed to study self-reported use of internal and external compensation strategies in older populations, was developed more than 20 years ago and does not reference digital technologies. Thus, measures of the tools that older adults use to support memory in daily life have not yet been updated for the digital age. Smart phones in particular may act as an effective and ubiquitous memory support device^18^, especially for certain kinds of memory tasks^12,17^. The more recent cognitive offloading literature has generally focused on physical tools in the environment, such as marking item locations^10^ or rotating a map^19^, rather than offloading to more everyday, ever-present digital tools (but see Finley et al.)^17^. Research that has focused on digital memory tools, examining the effects of photo taking and other digital storage tools on episodic memory performance, has provided mixed results and often not adequately matched how people use digital tools in daily life^15^. Our first research aim was to understand the uptake and impact of digital memory compensation strategies. Second, there has been limited direct comparison of the strategies reported by younger versus older adults and how these might be similar or different. Memory compensation is assumed to be a feature of adapting to age-related changes in cognitive capacity, but alternatively, the literature on cognitive offloading suggests that strategic memory compensation may be a lifelong phenomenon when confronted with cognitively challenging tasks.

### The current study

In the current research, we addressed these key gaps. We asked both younger and older participants to report the tools and strategies, internal and external, technological and non-technological, that they use to support different kinds of cognitive tasks. We classified the reported compensation strategies and examined which populations (in terms of age, gender, and attitudes to technology) use different kinds of support tools for different kinds of memory tasks.

## Method

### Participants

Participants were 228 younger adults (aged 17–25; *M* = 19.69, *SD* = 2.19) and 119 older adults (aged 60–89; *M* = 69.18, *SD* = 6.32). Participants identified their gender as 328 women, 85 men, and 3 other genders. Pre-determined sample size was at least 100 in each group, and a time-based stopping rule was employed prior to any data coding or analysis. The younger adults were recruited from a Psychology student research participation pool at Western Sydney University, and the older adults were recruited from The MARCS Institute for Brain, Behaviour and Development AgeLab Participant Registry. Ethics approval was provided by Western Sydney University Human Research Ethics Committee prior to participant recruitment (approval number H14215). All methods were performed in accordance with the relevant guidelines and regulations as specified in the Australian Code for the Responsible Conduct of Research. All participants provided informed consent. Data were collected between September and December 2021. This study was not preregistered.

### Measures

#### Memory compensation task questionnaire

We created a questionnaire based on the 20 different memory tasks referenced in the Memory Compensation Questionnaire^3^. The original questionnaire asks participants to rate the extent to which they use particular strategies for specific everyday memory tasks (e.g. “If you want to remember an appointment, do you write it down in a diary?”). For the current research, we generated an open-ended question for each different everyday memory task (e.g. “If you want to remember an appointment, how do you remember it?”). The full set of items is included in the Appendix.

#### Digital attitudes questionnaire—ability subscale (DAQ)^20^

The DAQ Ability Subscale includes 5 items that measure an individual’s relationship with and attitudes towards technology on a 5-point Likert scale ranging from 1 = *strongly disagree* to 5 = *strongly agree*. Questions related to notions of control, enthusiasm, learning, and confidence. Items are “Computers and technology give me more control over my life”, “I am interested in being able to access the internet wherever I am”, “I go out of my way to learn everything I can about new technology”, “I find technology is changing so fast, it’s difficult to keep up with it”, and “I keep my computer up to date with security software”.

### Procedure

Participants signed up online, were screened for eligibility, and were sent the Qualtrics survey link. The first page included study information and a consent form to digitally sign. Participants were informed that if they did not want to continue the study, they would be able to close their browser window and terminate their participation without consequence. Participants completed the survey in their own time and took approximately 30 min. Participants had to make a valid response to each item before moving to the next (i.e. type something into the free response box). On completion, older adults received a $10 electronic gift card, and students received course credit.

### Coding

For the free response data on the Memory Compensation Task Questionnaire, we developed a coding system to classify responses according to domains of interest. For each reported strategy, we distinguished whether it was “external” (relying on something outside the individual) or “internal” (relying on an individual’s brain or mind). For “external” strategies, we further coded them into: digital, physical, social, environmental, or indistinct (where we could not determine the external strategy type). For the internal strategies, we further coded them into time, effort, using a mnemonic strategy, or indistinct (where we could not determine the internal strategy type). All responses were scored by two independent coders. Initial agreement was 85.36%. Discrepancies were discussed to create a final set of codes for analysis. Responses were allowed to have more than one code where multiple strategies were mentioned. Table 1 presents details and examples of the coding categories.

**Table 1:** Coding categories with codebook definitions and examples.

	Definition	Examples
External		
Digital	The use of digital resources to assist remembering	“I write a list on my phone”; “I record it”; “I set a reminder on the TV”; “Google calendar it”
Physical	The use of physical resources or objects to assist remembering	“Write it in my diary”; “Fold the top corner of the page”; “Cut it out and keep it”
Social	Asking other people or relying on others for assistance	“My wife reminds me”; “Ask someone”; “Tell my parents to give me the mail when they are done so I do not forget”; “Tell other people constantly so my brain remembers”
Environmental	Using the intentional arrangement of the environment to assist remembering	“I place it near the front door”; “I keep things organised”; “I try and leave labels on what shelf contains what”; “I put it where I always do”
Indistinct	An external strategy that cannot be classified for certain as one of these subcategories (particularly when it was ambiguous about whether it was digital/physical)	“I add it to my calendar”; “Keep a copy”; “I write it down”; “Highlight, take notes”
Internal		
Time	Purposeful increase in encoding time e.g. through repetition	“Read it a few times”; “I always forget people’s names and have to ask multiple times until it sticks”; “Repeat it over in my head”
Effort	An explicit increase in effort or reference to an effortful intention to remember	“I try to memorize it the best I can”; “Focus when they are telling me it so I don’t forget”; “I actively listen”
Mnemonic	A complex cognitive strategy used to remember that goes beyond the stimulus itself	“Trace back my steps, looking back to where I last had it; “Visual presentation in my head”; “Try to remember a mutual friend then name pops up”
Indistinct	Ambiguous strategies that could not be classified into these types	“I remind myself to look at the clock often”; “I usually remember these kinds of things by heart”; “I automatically remember it”

## Results

### Age differences in memory compensation strategies

**Figure 1:**
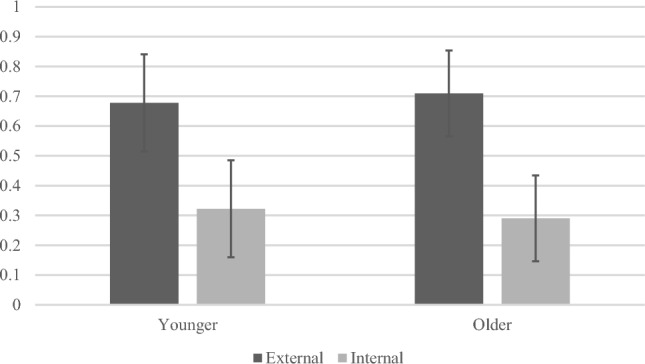
Proportion of external vs. internal memory compensation strategies reported by younger and older adults. Values are means, error bars are standard deviations.

Our first question was whether younger and older adults differed in their use of external and internal strategies. For each person, we scored the number and proportion of mentioned strategies that were external vs. internal. Across the 20 everyday memory tasks, older adults (*M* = 24.08, *SD* = 4.50) mentioned more strategies on average than younger adults (*M* = 22.42, *SD* = 4.40), *t*(345) = 3.32, *p* = 0.001. When examining the distribution of responses that were external vs. internal strategies, younger and older adults both reported a similarly high proportion of external strategies with no significant age difference, *t*(343) = 1.79, *p* = 0.074 (see Fig. 1). The vast majority of listed compensation strategies were coded as “external” across task types for both age groups.

Our second question focused on the different types of external strategies, particularly digital and physical compensation tools. For both younger and older adults, we calculated the distribution of their external tools across 5 categories: digital, physical, social, environmental, and indistinct (see Table 2). We used a 2 (age group) × 5 (external tool type) ANOVA to compare proportions. This analysis yielded a main effect of type, moderated by an interaction between age and type, *F*(4,340) = 11.11, *p* < 0.001, η_p_^2^ = 0.03. We followed this up with age comparisons on each tool type, with a Bonferroni correction for multiple comparisons (α < 0.01). Overall, digital tools were the most commonly reported technology across age groups (see Table 2). Younger adults reported a higher proportion of digital tools than older adults, *t*(343) = 3.62, *p* < 0.001, while older adults reported a higher proportion of physical tools than younger adults, *t*(343) = 5.38, *p* < 0.001. Younger adults reported more social strategies than older adults, *t*(343) = 2.87, *p* = 0.004, while older adults reported more environmental strategies, *t*(343) = 3.69, *p* < 0.001. Finally, younger and older adults had a similar number of responses that could not be classified into digital vs. physical, *t*(343) = 0.09, *p* = 0.924, and these indistinct responses were relatively common, making up about one third of responses.

**Table 2:** Percentage of younger and older adults’ external and internal memory compensation strategies coded into each subcategory.

	Younger	Older
External		
Digital	42.74 (22.59)	33.45 (22.70)
Physical	15.83 (11.93)	24.80 (16.02)
Social	5.90 (8.83)	3.69 (5.39)
Environmental	5.25 (6.49)	7.99 (6.65)
Indistinct	30.29 (22.42)	30.07 (19.46)
Internal		
Time	29.77 (26.42)	21.41 (23.14)
Effort	5.69 (10.12)	4.64 (9.26)
Mnemonic	26.05 (24.96)	27.72 (28.69)
Indistinct	36.50 (31.72)	47.80 (34.34)

Values are mean proportions with standard deviations in parentheses.

We also examined whether attitudes to technology were related to the use of digital technology as a memory compensation aid. A Pearson correlation coefficient was computed to assess the linear relationship between digital technology use and DAQ score, for younger and older adults separately. There was no significant correlation evident for younger adults *r*(226) = 0.097, *p* = 0.145. However there was a significant positive correlation between the two variables for older adults, *r*(117) = 0.376, *p* ≤ 0.001. This suggests that older adults with a more positive attitude towards technology were more likely to incorporate digital technology as a memory compensation strategy than older adults that had a more negative attitude towards technology. In contrast younger adults were just as likely to use digital technology regardless of their attitudes.

### Different strategies for different tasks

We included 20 different memory tasks in our survey, based on the exemplar everyday tasks employed in the Memory Compensation Questionnaire. We classified these tasks as involving either prospective memory (remembering to complete an intention in the future; e.g. When you want to remember an important appointment, what do you do to remind yourself?) or retrospective memory (bringing to mind a past event; e.g. When you want to remember the name of a particular person you are introduced to, how do you remember it?). We examined whether external vs. internal strategies were differentially employed for the different task types by conducting a 2 (age group: younger vs. older) × 2 (memory type: prospective vs. retrospective) mixed ANOVA on proportion-external scores. This analysis yielded only a main effect for memory task type, *F*(1,343) = 584.45, *p* < 0.001, η_p_^2^ = 0.63. Across age groups, prospective memory tasks had a higher percentage of external compensation strategies (*M* = 85.10, *SD* = 15.70) than retrospective memory tasks, which were more evenly split between external and internal strategies (*M* = 58.19, *SD* = 19.95). These data suggest that the type of memory task influences the type of strategy employed by both younger and older adults and that external memory compensation strategies are more heavily employed for prospective memory tasks.

### Gender differences

Prior research has suggested gender differences in Memory Compensation Strategies. We conducted a 2 (gender: male vs. female) × 2 (age group: younger vs. older) ANOVA on proportion-external scores. This analysis yielded main effects of gender, *F*(1,343) = 9.49, *p* = 0.002, η_p_^2^ = 0.03, and age group, *F*(1,343) = 8.73, *p* = 0.003, η_p_^2^ = 0.03, moderated by a significant interaction between them, *F*(1,343) = 4.39, *p* = 0.037, η_p_^2^ = 0.01. Follow-up pairwise comparisons suggested that in the younger age group, women (*M* = 69.89, *SD* = 15.60) reported a higher proportion of external strategies than men (M = 59.56, *SD* = 15.79), *t*(222) = 3.78, *p* < 0.001. In contrast in the older age group, both women (*M* = 71.60, *SD* = 13.87) and men (*M* = 69.64, *SD* = 15.56) reported a similarly high proportion of external strategies, *t*(112) = 0.70, *p* = 0.488.

## Discussion

Our findings indicate a high prevalence of external memory compensation strategy use by both younger and older adults. When provided with open ended questions about different everyday memory tasks (as opposed to frequency rating scales), older adults reported more strategies overall than younger adults, and both younger and older adults reported a preponderance of external memory compensation strategies. Although both younger and older adults reported similarly high levels of external compensation strategies, digital and social strategies were more common for younger adults and physical and environmental strategies were more common for older adults. Digital technology use by older adults was also more common if the older adults had a positive attitude towards technology. External strategies were more commonly reported for prospective than retrospective memory tasks, and less commonly reported by younger men.

These findings suggest the importance of external memory compensation strategies across age groups and tasks. The majority of strategies mentioned were external, although this varied by task and gender. Consistent with the broader literature on cognitive offloading^8^, the adoption of memory compensation strategies is not a phenomenon specific to an older or cognitively impaired population. Instead, our results demonstrate that younger people also compensate for challenges in completing everyday memory tasks. Theories of compensation suggest that compensation strategies are rapidly and temporarily employed when people perceive that their performance on a particular task may be less than they need or want^1,5^. Our results suggest that we encounter such needs across the lifespan, particular in meeting the prospective memory demands of our day-to-day life.

The high prevalence of external strategies across age groups also suggests a potential difference in findings between our methodology of asking people to freely report memory compensation strategies compared to providing people with compensation strategies and asking them to rate frequency of use (as in the Memory Compensation Questionnaire^3^). When people need to freely generate strategies used to accomplish daily tasks, they are more likely to generate external than internal strategies across both retrospective and prospective memory tasks (see also Finley et al.)^17^, although the particular preference for external strategies for prospective memory tasks converges with findings based on frequency ratings^12^. It is possible that with the uptake of digital technology, people have increased the extent to which they rely on external compared to internal resources over the last two decades, but further research is needed to examine the alignment of strategies reported via different methods.

Although younger adults reported more digital memory compensation strategies than older adults, both groups were more likely to report digital compared to physical tools. Thus, our results also speak to high rates of uptaking digital technology as a memory tool by both younger and older adults. Although older adults have sometimes been stereotyped as less technologically capable than younger adults^21^, digital memory compensations tools were the most heavily relied upon strategy for both younger and older adults. This finding suggests rapid uptake of digital technology among older adults and the potential role of digital technology in enhancing memory function in daily life for people who are older and those experiencing cognitive decline^18,22,23^. Moreover, those who were confident with technology and had a positive attitude towards it were more likely to use digital compensation tools. Therefore, combatting ageist stereotypes and enhancing older people’s perceptions of themselves as capable technology users may increase the extent to which people benefit from readily available digital tools. Current questionnaire methods for measuring the extent to which people engage in memory compensation strategies do not yet account for the uptake of digital technology within this older population, representing a key avenue for future research.

Overall, our results suggest that both younger and older adults engage in strategic and deliberate memory compensation strategies to complete day-to-day memory tasks. These strategies are most frequently external, especially for prospective memory tasks^12^, although less frequently for younger men. Importantly, these strategies frequently involve digital technology, for both younger and older adults, although there were some age differences in rates of reported strategy use. Caution is needed, given our study was conducted online and therefore included digitally-literate older adults. More socially vulnerable groups may not benefit to the same extent from technology that can support everyday memory function. Nevertheless, new measures of memory compensation are needed to capture the ubiquity of digital memory tools in daily life, in order to better represent the strategies used across populations. Moreover, combatting ageist stereotypes about technology use may increase the uptake and benefit of digital memory tools for older people.

## Supplementary Information

Supplementary Information.

## Data Availability

The data and associated explanatory documents can be accessed at https://osf.io/bjsqg/.
