# Supplementary material for: Memory compensation strategies in everyday life: similarities and differences between younger and older adults
Source: Sci Rep. 2023 May 24;13:8404. doi: 10.1038/s41598-023-34815-3 (PMC10209053; doi:10.1038/s41598-023-34815-3)
Supplement: Supplementary file 1 — Supplementary Information. [file 41598_2023_34815_MOESM1_ESM.docx]

**Appendix**

1. How do you remember what you need to buy when you go shopping? (prospective)
2. When you want to remember what someone is saying (e.g. an important conversation), how do you remind yourself? (retrospective)
3. When you want to remember an important appointment, what do you do to remind yourself? (prospective)
4. When you want to remember a story, what do you do to remember it? (retrospective)
5. When you are reading a book, how do you remember where you stopped reading last time? (retrospective)
6. When an interesting T.V. program is going to be on in the next few days, how do you remember to watch it? (prospective)
7. When you want to remember a newspaper article, what do you do to help you remember? (retrospective)
8. When you want to remember an upcoming event, such as a social event, how do you remember it? (prospective)
9. When you want to remember the name of a particular person you are introduced to, how do you remember it? (retrospective)
10. When you are reading something that really interests you (and that you want to remember), what do you do to remind yourself? (retrospective)
11. When you want to remember a conversation, how do you remember it? (retrospective)
12. When you are going to start a trip in the future, what do you do to remind yourself? (prospective)
13. When you want to remember where you put things, how do you remember it? (retrospective)
14. When you want to remember what to bring with you when you are going out, what do you do to remind yourself? (prospective)
15. When you want to remember appointments (for example, with the hairdresser or the dentist), what do you do to remind yourself? (prospective)
16. Before an important day, how do you remind yourself about the things you have to do to prepare? (prospective)
17. When you want to remember telephone numbers, how do you remember them? (retrospective)
18. When you want to remember something that happened on a particular day, what do you do in order to help you remember? (retrospective)
19. How do you remember when it is someone’s birthday, so that you can send them a card or greeting? (prospective)
20. When you want to remember a fact, like the name of a city, what do you do to help you remember? (retrospective)
